# Development of a Photocrosslinked Methacrylated Silk Fibroin Hydrogel Incorporating Hesperidin-Loaded Liposomes for Wound Dressing Applications

**DOI:** 10.3390/gels12100894

**Published:** 2026-10-02

**Authors:** Di Wang, Jiahua Li, Yongxia Wang, Zhiyuan Bao, Wanfu Yue

**Affiliations:** College of Animal Sciences & Technology, Zhejiang A&F University , Hangzhou 311300, China

**Keywords:** methacrylated silk fibroin, hesperidin-loaded liposomes, photocrosslinked hydrogel, Antioxidant activity, wound dressing

## Abstract

Hesperidin (HES) is a natural flavonoid with antioxidant activity; however, its poor aqueous solubility and low bioavailability limit its biomedical applications. In this study, HES-loaded liposomes (HES-LPs) were prepared by thin-film hydration and incorporated into a methacrylated silk fibroin (SilMA) hydrogel to construct a photocrosslinked composite hydrogel for potential wound dressing applications. Successful methacrylation of SilMA was confirmed by ^1^H nuclear magnetic resonance (^1^H NMR) spectroscopy. HES-LPs exhibited a mean particle size of 87.13 ± 0.84 nm, a zeta potential of −3.80 ± 0.49 mV, an encapsulation efficiency of 72.48%, and a drug loading of 4.62%. The composite hydrogel exhibited good antioxidant activity in 2,2-diphenyl-1-picrylhydrazyl (DPPH) and 2,2′-azino-bis(3-ethylbenzothiazoline-6-sulfonic acid) (ABTS) assays. It also exhibited sustained HES release, good cytocompatibility, and enhanced L929 fibroblast migration. Overall, the composite hydrogel represents a candidate wound-dressing platform supported by preliminary in vitro evidence.

## 1. Introduction

The skin, the body’s largest organ, serves as a critical barrier against environmental insults, helps maintain local and systemic physiological homeostasis, and limits transepidermal water loss [1]. Following injury, the body rapidly initiates a coordinated cascade of biological events—including hemostasis, inflammation, proliferation, and tissue remodeling—to restore the structure and function of the damaged tissue [2]. During wound healing, reactive oxygen species (ROS) exert dual effects. At moderate levels, ROS contribute to inflammatory responses, cellular signaling, tissue repair, and antimicrobial defense at the wound site [3]. However, excessive and sustained ROS production can overwhelm the body’s antioxidant capacity, resulting in oxidative stress and damage to cell membranes, proteins, and the extracellular matrix [4]. This oxidative imbalance can impair the function of key reparative cells, such as fibroblasts, prolong the inflammatory phase, and ultimately delay tissue regeneration and wound healing [5].

Hydrogels have good water retention and a three-dimensional network structure, making them suitable as carriers for local drug delivery, and they can help extend the action time of active ingredients at the wound site to some extent [6]^.^ By incorporating liposomes into the hydrogel network, the liposomes can act as carriers for the active ingredients, while the hydrogel provides local 3D support, which helps improve the stability of the active substances and regulate their release process [7]. So, combining liposomes with hydrogels offers a practical way to achieve sustained local delivery of active ingredients to wounds.

Natural polymer materials like silk fibroin (SF) have attracted a lot of attention in biomedical applications because of their excellent biocompatibility, degradability, and tunable mechanical properties [8]. By introducing methacryloyl groups onto the silk fi-broin molecular chains, it can be converted into methacrylated silk fibroin [9]. SilMA can be photo-crosslinked under mild conditions to quickly form a stable 3D network, which improves the gelation efficiency and structural controllability of the material [10]. At the same time, SilMA still retains the good biocompatibility of silk fibroin, providing a solid foundation for its use as a wound dressing material [11].

Hesperidin (HES), a naturally occurring citrus flavonoid, has attracted considerable attention owing to its potent antioxidant activity [12]. HES can effectively scavenge reactive oxygen species (ROS) and alleviate oxidative stress, thereby helping to maintain redox homeostasis and creating a favorable microenvironment for tissue re-pair [13,14]. In addition, its anti-inflammatory properties may further contribute to the regulation of the inflammatory response during wound healing [15]. However, the practical application of HES is largely limited by its extremely poor aqueous solubility [16], which results in insufficient dissolution and consequently low bioavailability, thereby restricting its effective utilization in biomedical applications [17]. Liposomal encapsulation provides a promising strategy to overcome these limitations by improving the aqueous dispersion, stability, and delivery efficiency of poorly water-soluble HES [18]. However, the combination of HES-loaded liposomes with SilMA hydrogels for sustained delivery and antioxidant functionality has not been sufficiently explored.

In this study, HES-loaded liposomes (HES-LPs) were incorporated into a photocrosslinked SilMA network to construct a composite hydrogel with potential as a candidate wound-dressing platform. By combining the structural and biocompatible properties of SilMA with the antioxidant activity of HES, the resulting hydrogel was designed to provide a favorable microenvironment and localized delivery of the bioactive compound. Its physicochemical properties, drug-release behavior, antioxidant activity, and in vitro biocompatibility were systematically evaluated, together with its potential to promote fibroblast migration. This work provides a potential strategy for incorporating poorly water-soluble natural bioactive compounds into protein-based hydrogels for the development of multifunctional wound dressings.

## 2. Results and Discussion

### 2.1. Characterization of Hesperidin-Loaded Liposomes

**Figure 1:**
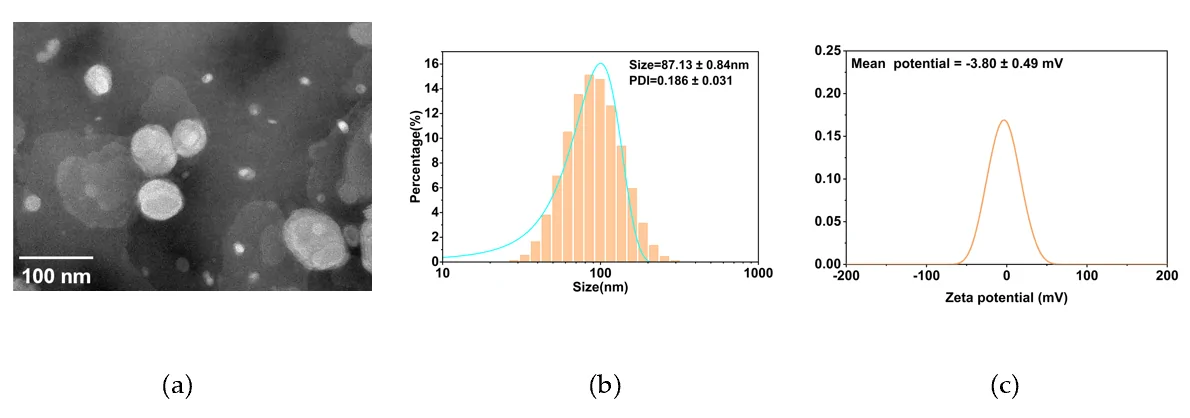
Characterization of Hesperidin-Loaded Liposomes (**a**) Transmission electron microscopy (TEM) image of HES-LPs at 100 k magnification (**a**); (**b**) Particle size distribution of HES-LPs; (**c**) Zeta potential of HES-LPs.

The morphology and physicochemical properties of the prepared HES-LPs were characterized by transmission electron microscopy (TEM), particle size distribution, and zeta potential analysis. As shown in Figure 1a, the HES-LPs exhibited predominantly spherical or near-spherical structures with relatively good dispersion, consistent with the typical morphology of liposomal vesicles [19].The particle size distribution of the HES-LPs is shown in Figure 1b. The particles were mainly distributed in the nanoscale range, with a mean particle size of 87.13 ± 0.84 nm and a PDI of 0.186 ± 0.031, indicating a relatively narrow particle-size distribution. This particle size was comparable to that reported for some hesperidin-loaded lipid-based nanocarriers, including lipid–polymer hybrid nanoparticles and solid lipid nanoparticles [20,21]. The zeta potential of the HES-LPs was −3.80 ± 0.49 mV (Figure 1c). The relatively low absolute zeta potential suggests that electrostatic repulsion may provide limited stabilization against particle aggregation. However, colloidal stability is also influenced by the composition and surface properties of liposomes. In particular, the DSPE-mPEG component in the formulation may provide additional steric stabilization, which could partially compensate for the relatively weak electrostatic repulsion. Overall, the TEM morphology, nanoscale particle size, and relatively low PDI supported the formation of a relatively uniform HES-LP system and provided a basis for its subsequent incorporation into the SilMA hydrogel network.

The encapsulation efficiency (EE) and drug loading content (DL) of the HES-LPs were determined to assess the incorporation of hesperidin into the liposomal formulation. As shown in Table 1, the HES-LPs exhibited an EE of 72.48% and a DL of 4.61%. The obtained EE falls within the range reported for related hesperidin-loaded lipid-based nanocarriers [20,21]. Nevertheless, direct comparisons should be interpreted with caution, as EE and DL are strongly influenced by the lipid composition, drug-to-lipid ratio, separation procedure, and analytical method employed. The EE value indicates that a substantial proportion of the initially added hesperidin was retained in the liposomal fraction after removal of the non-encapsulated drug, whereas the DL value represents the amount of hesperidin incorporated relative to the total mass of the recovered drug-loaded formulation. These results demonstrate the successful incorporation of hesperidin into the liposomal carrier and provide a basis for the subsequent integration of HES-LPs into the SilMA hydrogel network.

### 2.2. ^1^HNMR Characterization of SilMA

**Figure 2:**
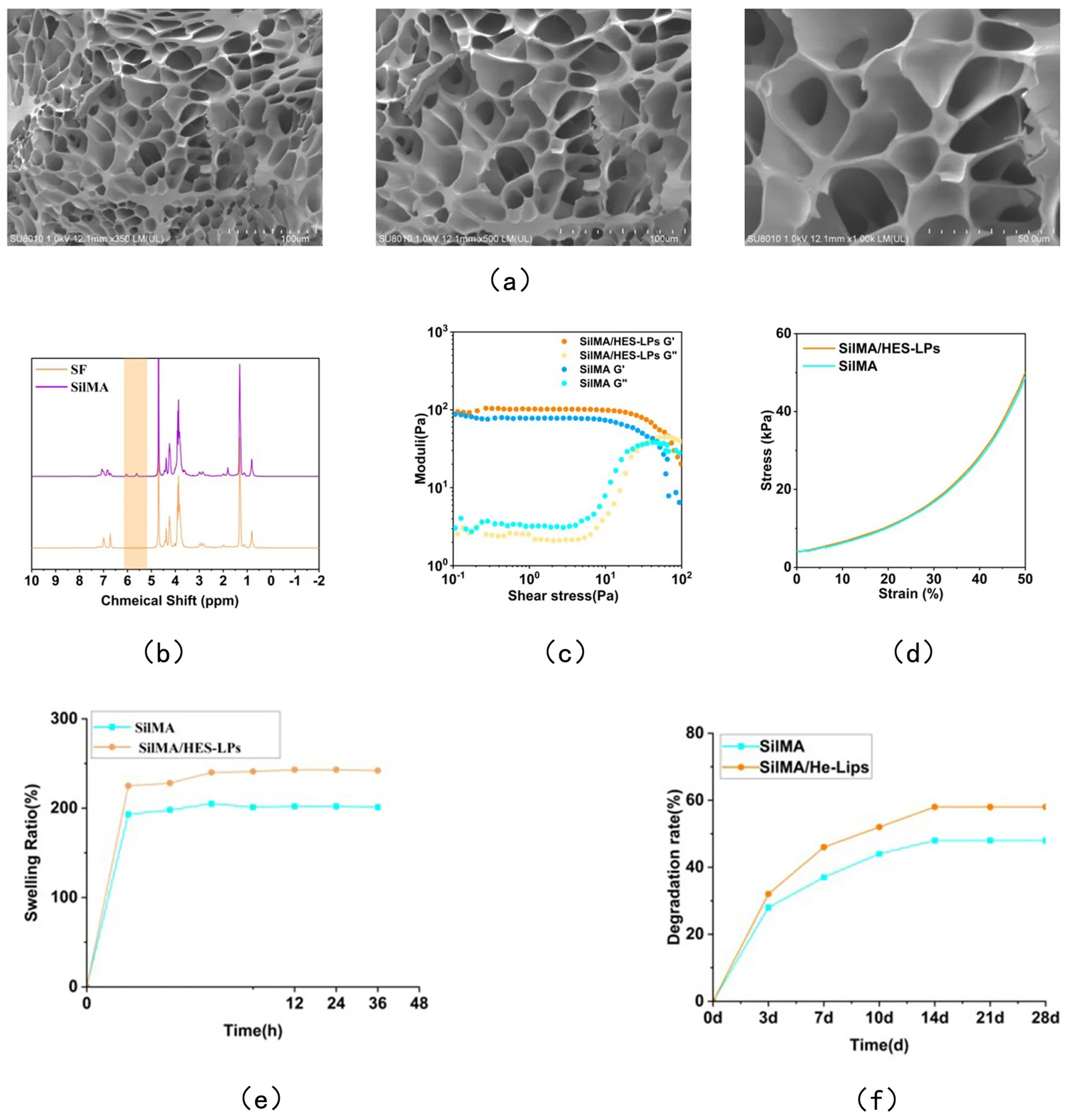
Characterization of SilMA and Physicochemical and mechanical properties of the hydrogels. (**a**) SEM images showing the internal microstructure of the SilMA/HES-LPs hydrogels at different magnifications. (**b**) ^1^H NMR characterization of SilMA. The characteristic resonances associated with the introduced methacrylate groups are highlighted in orange. (**c**) Rheological properties of the hydrogels. (**d**) Stress–strain curves of the hydrogels. (**e**) Swelling behavior of the hydrogels. (**f**) Degradation behavior of the hydrogels.

The chemical structures of pristine silk fibroin (SF) and methacrylated silk fibroin (SilMA) were investigated by ^1^H NMR spectroscopy. As shown in Figure 2b, pristine SF displayed the characteristic proton resonances associated with its polypeptide backbone and amino acid side chains. Following modification with glycidyl methacrylate (GMA), additional resonances appeared in the ^1^H NMR spectrum of SilMA. These newly observed signals were assigned to the methacrylate moieties introduced through the reaction between SF and GMA, and were consistent with previously reported methacrylated silk fibroin structures [22,23]. The characteristic resonances of the SF backbone remained detectable after methacrylation, suggesting that the major structural features of SF were preserved under the modification conditions. The incorporation of methacrylate groups rendered the modified SF amenable to photoinitiated crosslinking, thereby providing the required chemical functionality for the subsequent fabrication of SilMA-based hydrogels [22,24].

### 2.3. Characterization and Functional Properties of Composite Hydrogels

#### 2.3.1. Physicochemical and Mechanical Properties

The microstructure of the hydrogels was first examined by scanning electron microscopy (SEM). As shown in Figure 2a, SilMA/HES-LPs hydrogels exhibited interconnected porous structures, which may facilitate water penetration and the diffusion of incorporated substances. Similar interconnected porous architectures have been reported for silk fibroin-based and methacrylated silk fibroin hydrogels [23,25].

The rheological properties of the hydrogels were subsequently evaluated to investigate their viscoelastic behavior. As shown in Figure 2c, both hydrogels exhibited a low-stress linear viscoelastic region (LVR), within which G′ remained higher than G″, indicating predominantly elastic or solid-like behavior and suggesting the presence of an interconnected hydrogel network [22,24]. As the applied shear stress increased beyond the LVR, G′ gradually decreased, accompanied by an increase in G″, indicating progressive disruption of the hydrogel network and the onset of stress-induced yielding. The similar stress-dependent rheological responses of the two formulations suggest that the incorporation of HES-LPs did not substantially alter the overall viscoelastic behavior of the SilMA network.

The mechanical behavior of the hydrogels was further evaluated by stress–strain analysis (Figure 2d). Both hydrogels exhibited a progressive increase in stress with increasing strain and reached a stress of approximately 50 kPa at 50% strain. The close similarity between the two stress–strain curves suggests that the incorporation of HES-LPs did not substantially affect the bulk mechanical response of the SilMA hydrogel. This behavior may be attributed to the SilMA network remaining the primary load-bearing framework of the composite hydrogel. Similar rheological characteristics has been reported for SilMA-based hydrogel systems, in which the polymer network plays a dominant role in determining the mechanical properties [22,24].

The swelling behavior of the hydrogels is shown in Figure 2e. Both formulations exhibited rapid water uptake during the initial stage and gradually approached equilibrium after approximately 12 h. The equilibrium swelling ratios were approximately 200% for SilMA and 240% for SilMA/HES-LPs. The composite hydrogel exhibited a significantly higher swelling ratio at the tested time points. Similar swelling behavior has been reported for silk fibroin-based hydrogels, in which water uptake is closely associated with the hydrophilic groups and porous network structure of the hydrogel [26,27]. The increased swelling capacity of the SilMA/HES-LPs hydrogel may be related to structural changes induced by the incorporation of HES-LPs, which could facilitate water penetration and retention within the hydrogel network.

The degradation behavior of the hydrogels was monitored over 28 days (Figure 2f). Both formulations underwent gradual degradation over time, with a relatively faster decrease during the early stage. After 28 days, the cumulative mass loss of the SilMA and SilMA/HES-LPs hydrogels was approximately 48% and 58%, respectively. Previous studies have shown that the degradation behavior of silk fibroin-based hydrogels is influenced by factors such as network structure, crosslinking density, and water permeability [28,29]. The slightly greater degradation observed for the composite hydrogel may therefore be associated with its higher swelling capacity, which could facilitate water penetration and mass transport within the network [29].

Overall, incorporation of HES-LPs preserved the porous structure, viscoelasticity, and rheological properties of the SilMA hydrogel, while increasing its swelling capacity and slightly accelerating degradation. These properties suggest that Sil-MA/HES-LPs possesses suitable structural stability and water-management characteristics for its potential application as a local HES delivery system for wound dressing.

#### 2.3.2. Drug Release Behavior

**Figure 3:**
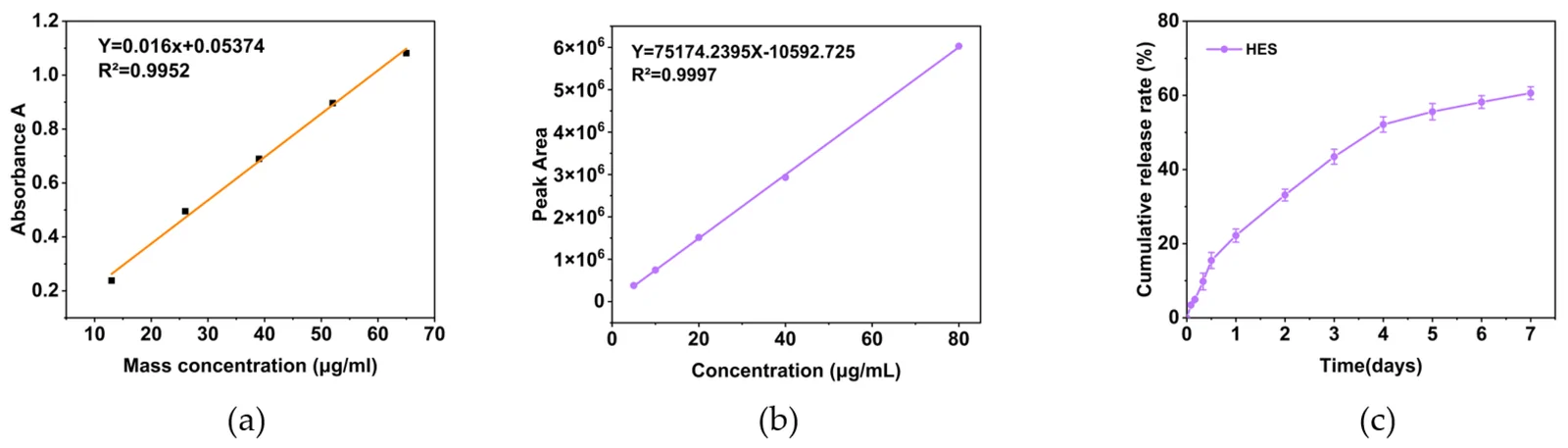
Drug release behavior of the SilMA hydrogel. (**a**) Standard calibration curve of HES measured by UV–Vis spectrophotometry. (**b**) Standard calibration curve of HES measured by HPLC. (**c**) In vitro cumulative release profile of HES from the SilMA/HES−LPs hydrogel.

The drug release behavior of the composite hydrogel was evaluated by HPLC. HES calibration curves were established using both UV–Vis spectrophotometry and HPLC. The UV–Vis calibration curve was used to determine the encapsulation efficiency and drug loading of HES-LPs, whereas the HPLC calibration curve was used for quantitative analysis of HES released from the hydrogel. As shown in Figure 3a, HES exhibited a good linear relationship between absorbance and concentration over the range of 10–70 μg/mL, with a regression equation of Y = 0.016X + 0.05374 and a high coefficient of determination (R^2^ = 0.9952). Similarly, the HPLC calibration curve showed excellent linearity over the concentration range of 0–80 μg/mL, with a regression equation of Y = 75,174.2395X − 10,592.725 and an R^2^ value of 0.9997 (Figure 3b).

As shown in Figure 3c, the SilMA/HES-LPs hydrogel exhibited sustained HES release over 7 days. Approximately 22% of the encapsulated HES was released during the first day, followed by a gradual increase to approximately 33% and 43% on days 2 and 3, respectively. From days 3 to 5, the cumulative release continued to increase, reaching approximately 55%. Thereafter, the release rate gradually decreased, with cumulative release reaching approximately 58% on day 6 and 61% on day 7. The cumulative release reached approximately 61% at the end of the 7-day period, indicating that HES release gradually approached a plateau. Similar sustained-release profiles have been reported for silk fibroin-based hydrogel systems, supporting the potential of SilMA as a matrix for controlled drug delivery [30,31]. In addition, liposome–hydrogel systems have been reported to prolong drug retention and modulate release through the combined effects of liposomal encapsulation and diffusion through the hydrogel network [32].

The observed release profile may be primarily associated with diffusion of HES through the hydrogel network, consistent with previously reported hydrogel-based drug delivery systems [30,31]. During the initial stage, HES located near the hydrogel surface or within readily accessible aqueous regions may diffuse into the surrounding medium more readily. As release proceeds, the increasing diffusion distance and resistance imposed by the hydrogel network progressively limit HES transport, resulting in a gradual reduction in the release rate. The relatively stable release observed at later time points further suggests that the SilMA network restricts HES diffusion and thereby contributes to prolonged release. Overall, the SilMA/HES-LPs hydrogel showed sustained HES release over 7 days, indicating its potential as a controlled-release platform for HES delivery.

#### 2.3.3. Antioxidant Activity of the Hydrogels

**Figure 4:**
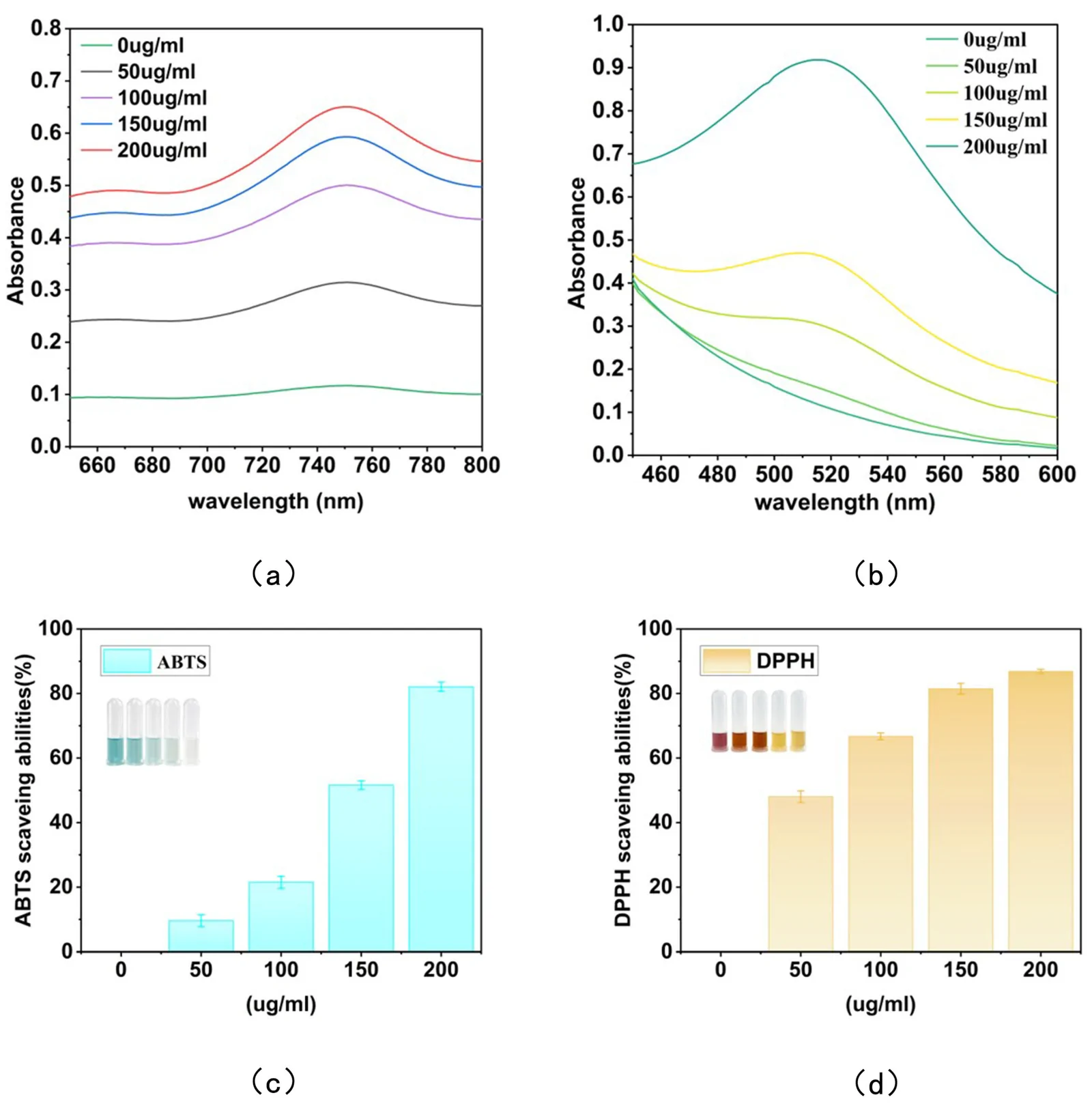
Antioxidant activity of composite hydrogel extracts containing different concentrations of hesperidin (50, 100, 150, and 200 μg/mL). (**a**) Absorbance of the ABTS radical solution. (**b**) Absorbance of the DPPH radical solution. (**c**) ABTS radical scavenging rate. (**d**) DPPH radical scavenging rate.

The antioxidant activity of the composite hydrogel extracts containing different concentrations of HES-LPs was evaluated using DPPH and ABTS radical scavenging assays. As shown in Figure 4a,b, the absorbance of the radical solutions varied with increasing HES-LPs concentration, indicating the antioxidant activity of the hydrogel extracts. The corresponding quantitative results are presented in Figure 4c,d. As the concentration increases, the ABTS and DPPH free radical scavenging rates gradually go up. This concentration-dependent antioxidant activity is consistent with the previously reported antioxidant properties of hesperidin [33,34].

The two antioxidant assays showed different scavenging efficiencies, particularly at relatively low HES-LPs concentrations. The DPPH radical scavenging rate was higher than the ABTS radical scavenging rate at the same HES-LPs concentration, whereas the difference between the two assays became less pronounced at higher concentrations. At 200 μg/mL, the scavenging rates of DPPH and ABTS radicals reached approximately 87% and 82%, respectively, indicating the strong antioxidant activity of the HES-LPs-containing composite hydrogel. Similar antioxidant activity has been reported for hesperidin-loaded delivery systems, suggesting that encapsulation can preserve the radical-scavenging activity of HES [35]. The concentration-dependent increase in radical scavenging activity suggests that the antioxidant properties of HES were retained after its encapsulation into liposomes and subsequent incorporation into the SilMA hydrogel network. The HES-LPs-containing composite hydrogel therefore exhibited effective free radical scavenging activity, which may help reduce oxidative stress in the wound microenvironment and support its potential application in wound repair [33,34].

### 2.4. Cytocompatibility and Cell Migration of the Composite Hydrogel

**Figure 5:**
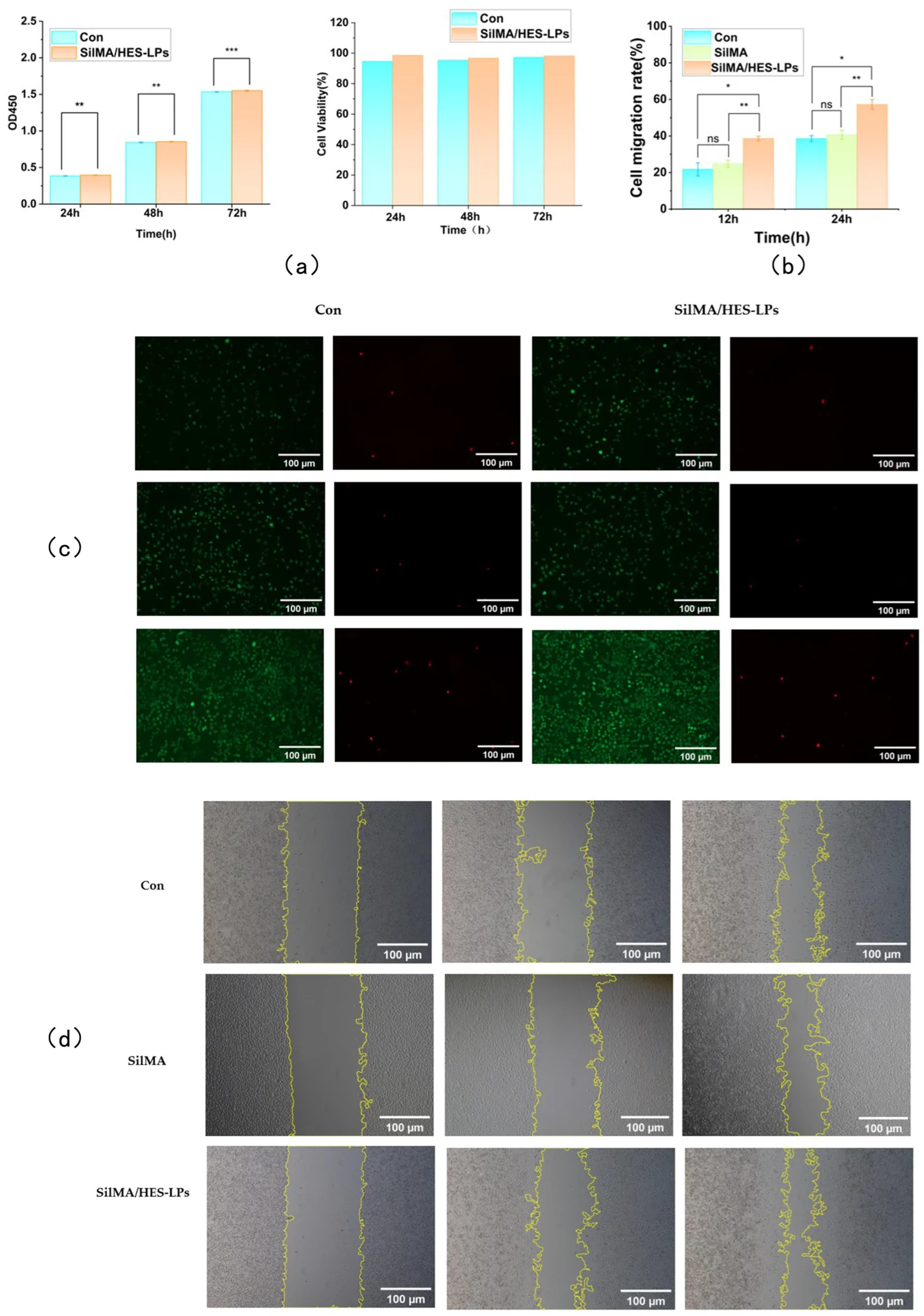
Cytocompatibility and cell migration of L929 cells in response to the composite hydrogels. (**a**) CCK-8 assay results showing the absorbance and corresponding cell viability of L929 cells cultured with extracts of the Con and SilMA/HES-LPs groups for 24, 48, and 72 h. (**b**) Quantitative analysis of L929 cell migration rates in the Con, SilMA, and SilMA/HES-LPs groups at 12 and 24 h in the scratch wound assay. (**c**) Representative fluorescence images of L929 cells cultured on the Con and SilMA/HES-LPs hydrogel samples for 24, 48, and 72 h and stained using a Calcein-AM/PI live/dead assay. Live cells are shown in green by Calcein-AM staining, whereas dead cells are shown in red by PI staining. (**d**) Representative images of the scratch wound assay showing L929 cell migration in the Con, SilMA, and SilMA/HES-LPs groups at 0, 12, and 24 h. Statistical significance for (**a**,**b**) was determined by two-way ANOVA followed by Tukey’s multiple-comparison test. * *p* < 0.05, ** *p* < 0.01, and *** *p* < 0.001; ns indicates no significant difference.

First, we used a CCK-8 assay based on extract solutions to check the cell compatibility of the SilMA/HES-LPs hydrogel. As shown in Figure 5a, the OD450 values for both the control group and the SilMA/HES-LPs group steadily increased from 24 h to 72 h, indicating continuous cell growth. The values between the two groups were comparable throughout the observation period, suggesting that the hydrogel extracts didn’t significantly inhibit cell proliferation. Consistently, the calculated cell viability stayed above 90% at all the tested time points. These results show that the SilMA/HES-LPs hydrogel has good cell compatibility, consistent with previous reports of the favorable cytocompatibility of silk fibroin- and SilMA-based biomaterials [36].

The AMPI live/dead cell staining assay further evaluated the effects of the hydrogel on cell viability and proliferation (Figure 5c). In both groups, mainly green fluorescent live cells were observed at 24, 48, and 72 h, with only a few red fluorescent dead cells. In the SilMA/HES-LPs group, the number and density of live cells gradually increased with prolonged culture time, and more live cells were observed at the corresponding time points compared to the control group. This result, together with the CCK-8 assay results, indicates that the SilMA/HES-LPs hydrogel not only exhibits low cytotoxicity but also provides a favorable microenvironment for cell proliferation. The enhanced cell proliferation in the experimental group may be related to the bioactivity of HES and the hydrated three-dimensional structure of the hydrogel, which may provide suitable conditions for cell growth [36].

Then, a scratch assay was used to evaluate the effect of the hydrogel on cell migration (Figure 5b,d). Compared with the control group, the SilMA-alone group showed similar cell migration rates at both 12 and 24 h, indicating that SilMA itself did not markedly enhance cell migration under the conditions tested. In contrast, the SilMA/HES-LPs group showed a more obvious reduction in scratch area at both 12 and 24 h. The corresponding cell migration rates of the SilMA/HES-LPs group were about 38% and 57% at 12 and 24 h, respectively, compared with approximately 22% and 39% in the control group. These results suggest that the enhanced cell migration observed with the composite hydrogel was associated with the incorporation of HES-LPs. The enhanced cell migration might be related to the bioactivity of hesperidin and the favorable microenvironment provided by the hydrogel. Previous studies have reported that silk fibroin-based biomaterials can support fibroblast migration and wound repair, while hesperidin has also been associated with biological activities relevant to tissue repair [37]. Overall, the SilMA/HES-LPs hydrogel showed good cytocompatibility and promoted fibroblast migration, suggesting that it may have potential for use as a wound dressing.

## 3. Conclusions

In this study, HES-loaded liposomes (HES-LPs) were successfully prepared and incorporated into a photocrosslinked SilMA hydrogel to form a composite hydrogel with potential as a candidate wound-dressing platform. The HES-LPs showed a mean particle size of 87.13 ± 0.85 nm and an encapsulation efficiency of 72.48%. The composite hydrogel exhibited a well-defined three-dimensional porous structure, sustained HES release, antioxidant activity, good cytocompatibility, and enhanced L929 fibroblast migration, indicating its potential for wound-healing applications. Currently, this study was mainly based on in vitro experiments, and in vivo wound-healing efficacy and practical wound-dressing performance were not evaluated. Future studies should further evaluate its performance in animal wound models.

## 4. Materials and Methods

### 4.1. Materials

HES was purchased from Shanghai Yuanye Bio-Technology Co., Ltd. (Shanghai, China). SF was extracted from commercially available silk. Glycidyl methacrylate (GMA) was obtained from TCI (Shanghai) Development Co., Ltd. (Shanghai, China) and used for the modification of SF. Lithium phenyl-2,4,6-trimethylbenzoylphosphinate (LAP) was purchased from Guangdong Yuxi Biotechnology Co., Ltd. (Foshan, China) and used as a photoinitiator for hydrogel photocrosslinking.

2,2-Diphenyl-1-picrylhydrazyl (DPPH) and 2,2′-azino-bis(3-ethylbenzothiazoline-6-sulfonic acid) (ABTS) were purchased from Shanghai Macklin Biochemical Technology Co., Ltd. (Shanghai, China). Phosphate-buffered saline (PBS) was obtained from [Supplier, Hangzhou, China]. All other chemicals and reagents were of analytical grade and used as received without further purification. Deionized water was used throughout the experiments.

L929 mouse fibroblast cells were purchased from Shanghai Fuheng Biotechnology Co., Ltd. (Shanghai, China). Dulbecco’s modified Eagle’s medium (DMEM) and fetal bovine serum (FBS) were obtained from Shanghai Fuheng Biotechnology Co., Ltd. (Shanghai, China). Cell Counting Kit-8 (CCK-8) and the Live/Dead Cell Viability Assay Kit were purchased from Shanghai Yeasen Biotechnology Co., Ltd. (Shanghai, China). All cell culture-related reagents were used according to the manufacturers’ instructions.

### 4.2. Preparation and Characterization of Hesperidin-Loaded Liposomes

HES-LPs were prepared by the thin-film hydration method. Briefly, 17 mg of lecithin, 2 mg of cholesterol, 5 mg of DSPE-mPEG, and 0.6 mg of hesperidin were accurately weighed and dissolved in a mixture of dichloromethane (20 mL) and methanol (10 mL). The mixture was sonicated to obtain a homogeneous solution and then transferred to a rotary evaporator. Dichloromethane was removed under reduced pressure at 40 °C and 150 rpm to form a uniform lipid film on the inner wall of the flask. The film was hydrated with 6 mL of PBS and sonicated for 5 min, followed by hydration at 37 °C for 2 h. The resulting suspension was sonicated using a probe sonicator in an ice bath for 2 min at 35% amplitude (3 s on and 1 s off). The obtained HES-LP suspension was concentrated by ultrafiltration and stored for further experiments.

EE and drug loading content (DL) of HES-LPs were determined by ultrafiltration centrifugation. Briefly, 1 mL of HES-LP suspension was transferred to an ultrafiltration tube (MWCO 4 kDa) and centrifuged at 8000 rpm for 20 min at 4 °C. Then, 200 μL of the filtrate was mixed with 200 μL of methanol, and the HES concentration was determined by UV–Vis spectrophotometry using a standard curve. The measured HES content was considered the amount of free HES. The EE and DL were calculated using the following equations, where (W*_HES,initial_*) represents the initial amount of hesperidin added during liposome preparation, and (W*_HES,free_*) represents the amount of free hesperidin measured in the filtrate. In the DL equation, (W*_HES,encapsulated_*) represents the amount of hesperidin encapsulated in the liposomes, and (W*_HES-LPs_*) represents the total mass of the HES-LPs. The measurements were performed in triplicate using three independent samples. EE and DL were determined using three independent samples.


$$EE(\%)=\frac{W_{HES,initial}-\;W_{HES,free}}{W_{HES,initial}}\times 100\%$$

$$DL(\%)=\frac{W_{HES,encapsulated}}{W_{HES\text{-}LPs}}\times 100\%$$

### 4.3. Preparation of SilMA and Composite Hydrogels

#### 4.3.1. Preparation of SilMA

SilMA was prepared according to a previously reported method with slight modifications. Briefly, 50 g of cocoons with the pupae removed were boiled twice in 1 L of 0.05 M Na_2_CO_3_ solution (5.3 g) at 100 °C for 30 min each time. The degummed silk fibroin (SF) was washed several times with distilled water and dried in an oven at 60 °C. Subsequently, 20 g of degummed SF was dissolved in 100 mL of 9.3 M LiBr solution at 60 °C with stirring for 1 h. After complete dissolution, the solution was centrifuged at 10,000 rpm for 5 min and filtered through Miracloth. Then, 12 mL of glycidyl methacrylate (GMA) was added to the SF solution, and the mixture was stirred at 1000 rpm and 60 °C for 8 h. After the reaction, the solution was centrifuged at 10,000 rpm for 5 min and filtered through Miracloth. The filtrate was dialyzed against distilled water using a dialysis membrane (MWCO 8–14 kDa) for 3–5 days to remove residual salts. Finally, the dialyzed solution was frozen at −80 °C and then lyophilized to obtain SilMA.

#### 4.3.2. Preparation of Composite Hydrogels

The composite hydrogels were prepared by photocrosslinking SilMA in the presence of HES-LPs. Briefly, LAP was dissolved in deionized water at a concentration of 0.25% (*w*/*v*) under light-protected conditions. The solution was heated in a water bath at 60 °C and shaken several times until the LAP was completely dissolved. SilMA was then dissolved in 1 mL of the 0.25% (*w*/*v*) LAP solution under light-protected conditions to obtain a 100 g/L SilMA solution. The mixture was heated at 60 °C and shaken several times until a homogeneous solution was obtained. Subsequently, 800 μL of HES-LPs was added and mixed thoroughly. The resulting precursor solution was exposed to 405 nm light for photocrosslinking. The hydrogel was formed within 15 s and designated as the SilMA/HES-LPs composite hydrogel.

#### 4.3.3. Material Characterization

The hydrogel samples were rapidly frozen in liquid nitrogen and then fractured. The fractured samples were subsequently freeze-dried for 48 h. The dried samples were mounted on sample stubs with the fractured surfaces facing upward and sputter-coated with a thin layer of gold (approximately 10 nm) using an ion sputter coater. The internal microstructure of the hydrogels was observed using a field-emission scanning electron microscope (FE-SEM, SU8010, Hitachi, Tokyo, Japan) at an accelerating voltage of 1.0 kV. Rheological characterization of the composite hydrogels was performed using a rotational rheometer (MCR 302, Anton Paar, Ostfildern, Germany) equipped with a 25 mm diameter parallel-plate geometry. The gap was set to 1 mm, and the measurements were conducted at 25 °C. An oscillatory stress sweep was performed over a shear stress range of 10^−1^–10^3^ Pa at a constant frequency of 1 Hz to record the storage modulus (G′) and loss modulus (G″) as a function of applied shear stress. The linear viscoelastic region (LVR) was identified from the stress sweep as the low-stress plateau region in which G′ and G″ remained approximately independent of the applied shear stress before the onset of a pronounced decrease in G′.

The swelling behavior of the hydrogels was evaluated by the gravimetric method. Briefly, the hydrogels were completely dried and accurately weighed to obtain the initial dry weight (M_0_). The dried hydrogel samples were immersed in excess PBS and allowed to swell in a constant-temperature water bath at 37 °C. At predetermined time points (1, 2, 4, 8, and 24 h), the samples were removed from the PBS, and excess water on the surface was gently removed using filter paper. The swollen samples were immediately weighed to obtain the wet weight (M_t_). The swelling ratio (SR) was calculated according to the following equation:

$$\mathrm{Swelling}\;\mathrm{Ratio}\;=\;\frac{M_{t}-M_{0}}{M_{0}}\times 100\%$$

The initial mass of the dried hydrogel samples was accurately weighed (W_0_), after which they were immersed in PBS containing 0.02% (*w*/*v*) NaN_3_ (as a bacteriostatic agent) and incubated at 37 °C in a constant-temperature shaker with oscillatory agitation at 60 rpm. The PBS solution was refreshed every 48 h. At predetermined time points (days 3, 7, 10, 14, 21 and 28), the samples were removed, rinsed with deionized water, freeze-dried, and weighed again to obtain the dry mass (W_t_). The percentage of remaining mass and the degradation rate were calculated according to Equation (2). Each group was tested in triplicate (*n* = 3).

$$\mathrm{Degradation}\;(100\%)=1-\frac{W_{t}}{W_{0}}\times 100\%$$

### 4.4. Drug Release, Antioxidant Activity, and In Vitro Biological Evaluation

#### 4.4.1. Establishment of Hesperidin Standard Curves by UV–Vis and HPLC

A HES standard curve was established to determine the HES concentration. Briefly, 6 mg of HES standard was accurately weighed and dissolved in 1 mL of DMSO to prepare a stock solution. The UV–Vis absorption spectrum was recorded from 200 to 400 nm using DMSO as the blank, and the maximum absorption wavelength of HES was determined to be 286 nm. The stock solution was then serially diluted to obtain HES standard solutions at concentrations of 13, 26, 39, 52, and 65 μg/mL. The absorbance of each solution was measured at 286 nm. A calibration curve was obtained by plotting absorbance (A) against HES concentration (C, μg/mL), followed by linear regression analysis.

An appropriate amount of HES reference standard was accurately weighed and dissolved in the mobile phase to prepare a stock solution. The stock solution was subsequently diluted with the mobile phase to obtain HES working solutions at concentrations of 5, 10, 20, 40, and 80 μg/mL. Each standard solution was injected into the HPLC system under the established chromatographic conditions, and the chromatographic peak of HES was detected at 283 nm. The peak area corresponding to HES was recorded for each concentration. A calibration curve was constructed by plotting HES concentration (C, μg/mL) on the *x*-axis against the corresponding peak area (A) on the *y*-axis. Linear regression analysis was performed to obtain the calibration equation.

#### 4.4.2. In Vitro Hesperidin Release from the Composite Hydrogel

The in vitro release of HES from the composite hydrogel was evaluated by the direct immersion method. Briefly, 1 mL of the HES-LPs-loaded composite hydrogel was immersed in PBS containing 0.5% (*w*/*v*) Tween 80. The hydrogel was completely submerged in the release medium during the release period.

At predetermined time points (2, 4, 8, and 12 h, and 1, 2, 3, 4, 5, 6, and 7 days), 0.5 mL of the release medium was withdrawn and immediately replaced with an equal volume of fresh release medium. The HES concentration in the collected samples was determined by HPLC according to the established chromatographic conditions and the HES calibration curve. The cumulative amount of HES released at each time point was calculated by taking into account the HES concentration in the collected samples and the volume replacement during sampling, as follows:

(1) $$M_{n}=C_{n}V_{0}+V_{s}\sum_{i=1}^{n-1}C_{i}$$

where M_n_ is the cumulative amount of HES released at the nth sampling time point (mg); C_n_ is the HES concentration in the release medium at the nth sampling time point (mg/mL); C_i_ is the HES concentration measured at the ith sampling time point (mg/mL); V_0_ is the total volume of the release medium; and V_s_ is the volume of the release medium withdrawn at each sampling time point. The cumulative HES release rate was calculated according to the following Equation (2):

(2) $$M_{n}=10C_{n}+\sum_{i=1}^{n-1}C_{i}$$

The cumulative drug release rate at the nth time point is calculated using Equation (3):

(3) $$Q_{n}=\frac{M_{n}}{M_{0}}\times 100\%$$

In the formula, Q_n_ is the cumulative drug release rate (%) at the nth time point; M_0_ is the initial amount of drug in the hydrogel sample, which in this experiment is set as 1 mg according to the theoretical input. The in vitro drug release curve is plotted with release time on the *x*-axis and cumulative drug release rate on the *y*-axis. Three parallel samples are set for each group, and the results are presented as the mean ± standard deviation.

#### 4.4.3. Antioxidant Activity

The antioxidant activity of the hydrogels was evaluated by DPPH and ABTS radical scavenging assays. Hydrogels containing HES-LPs at concentrations of 50, 100, 150, and 200 μg/mL were prepared, and the corresponding hydrogel extracts were collected for subsequent analysis.

For the DPPH assay, a 0.2 mM DPPH solution was prepared in ethanol. The hydrogel extracts were mixed with the DPPH solution and incubated in the dark. The absorbance of the mixture was measured at 516 nm using a UV–Vis spectrophotometer.

For the ABTS assay, the hydrogel extracts were mixed with diluted ABTS working solution and incubated in the dark. The absorbance was measured at 734 nm using a UV–Vis spectrophotometer.

The DPPH and ABTS radical scavenging rates were calculated using the following equations:

$$DPPH\;scavenging\;rate\;\left(\%\right)=\frac{A_{0}-A_{s}}{A_{0}}\times 100\%$$

$$ABTS\;scavenging\;rate\left(\%\right)=\frac{A_{1}-A_{s}}{A_{1}}\times 100\%$$

where A_0_ is the absorbance of the DPPH or ABTS solution without the hydrogel extract, and A_s_ is the absorbance of the corresponding solution after addition of the hydrogel extract.

#### 4.4.4. In Vitro Biological Evaluation

The cytocompatibility of the hydrogel was examined using its extracts following the procedures described in ISO 10993-5 [38]. Briefly, sterilized hydrogel specimens were transferred to complete culture medium at a surface area-to-medium ratio of 1 cm^2^/mL and maintained at 37 °C under 5% CO_2_ for 24 h. The resulting extracts were collected for subsequent cell assays. L929 cells were seeded into 96-well plates at 5 × 10^3^ cells per well and allowed to adhere for 24 h. The medium was subsequently replaced with 100 μL of either undiluted hydrogel extract, 50% diluted extract, or fresh complete medium serving as the negative control. Cell viability was assessed after 24, 48, and 72 h of exposure by adding 10 μL of CCK-8 reagent to each well and incubating for 2 h. Absorbance was recorded at 450 nm using a microplate reader. The value obtained for the negative control was defined as 100%, and the relative proliferation rates of the treatment groups were calculated accordingly. Three independent experiments were conducted, with six technical replicates included for each group (*n* = 3 biological replicates, *n* = 6 technical replicates).

For live/dead staining, sterilized circular hydrogel samples were placed at the bottom of 24-well plates. L929 cells were directly seeded onto the hydrogel surfaces at a density of 2 × 10^4^ cells per well and cultured for 24,48 and 72 h. The culture medium was then removed, and the samples were washed once with PBS. Each well was then treated with a staining solution containing 5 μM Calcein-AM and 15 μM propidium iodide (PI). Staining was carried out at 37 °C in the dark for 20 min. The stained samples were immediately examined under a fluorescence inverted microscope using a 10× objective. Calcein-AM fluorescence was used to identify viable cells, whereas PI fluorescence indicated dead cells. The assay was independently repeated three times.

For the scratch wound assay, L929 cells were seeded in 6-well plates at a high density of 5 × 10^5^ cells per well and cultured until a confluent monolayer was established. A straight scratch was created in the center of each cell monolayer using a sterile 200 μL pipette tip. The wells were gently washed twice with PBS to remove detached cells. The SilMA-alone and SilMA/HES-LPs groups were then supplemented with fresh medium containing 50% of the corresponding extract, while the control wells received fresh complete medium. Images of the scratched region were acquired immediately after scratching and at 12 and 24 h using an inverted microscope equipped with a 4× objective. The scratched area was quantified with ImageJ software (version 1.54), and the cell migration rate was determined according to Equation (1). Three independent experiments were performed, and at least three randomly selected fields were evaluated for each group.

$$\mathrm{Migration}\;\mathrm{Rate}=\frac{{Area}_{0h}-{Area}_{24h}}{{Area}_{0h}}\times 100\%$$

### 4.5. Statistical Analysis

All quantitative data are presented as the mean ± standard deviation (SD). Statistical analyses were performed using IBM SPSS Statistics 27.0.1. For datasets involving a single experimental factor, including ABTS and DPPH radical-scavenging activities, and cumulative HES release at different time points, one-way analysis of variance (ANOVA) followed by Tukey’s multiple-comparison test was used. For datasets involving both treatment group and time as experimental factors, including cell viability and scratch wound closure, two-way ANOVA followed by Tukey’s multiple-comparison test was used.

Technical replicates were used for physicochemical characterization, antioxidant assays, and other quantitative experiments, whereas biological replicates with technical replicates were used for cell-based experiments where applicable. A *p*-value of <0.05 was considered statistically significant. In the figures, * *p* < 0.05, ** *p* < 0.01, and *** *p* < 0.001; ns indicates no significant difference. Figures and graphs were generated using Origin 2024b.

## Figures and Tables

**Table 1:** Physicochemical characteristics of HES-LPs.

Name	Data
Size	87.13 ± 0.84 nm
PDI	0.186 ± 0.031
Zeta	−3.80 ± 0.49 mV
Encapsulation Efficiency(%)	72.48 ± 2.78
Drug loading (%)	4.62 ± 0.18

## Data Availability

The original contributions presented in this study are included in the article. Further inquiries can be directed to the corresponding author.

## Abbreviations

The following abbreviations are used in this manuscript:

SF	Silk Fibroin
SilMA	Methacrylated silk fibroin
Hes	Hesperidin.
HES-LPs	Hesperidin-loaded liposomes
^1^HNMR	Proton nuclear magnetic resonance spectroscopy
TEM	Transmission Electron Microscopy
DPPH	2,2-diphenyl-1-picrylhydrazyl
ABTS	2,2′-azino-bis(3-ethylbenzothiazoline-6-sulfonic acid)
ROS	Reactive Oxygen Species
CCK8	Cell Counting Kit-8
PBS	Phosphate-buffered saline
EE	Encapsulation Efficiency
DL	Drug Loading
GMA	Glycidyl methacrylate
